# Supplementary figures and images for: COVID-19 vaccine hesitancy and resistance: Correlates in a nationally representative longitudinal survey of the Australian population
Source: PLoS One. 2021 Mar 24;16(3):e0248892. doi: 10.1371/journal.pone.0248892 (PMC7990228; doi:10.1371/journal.pone.0248892)

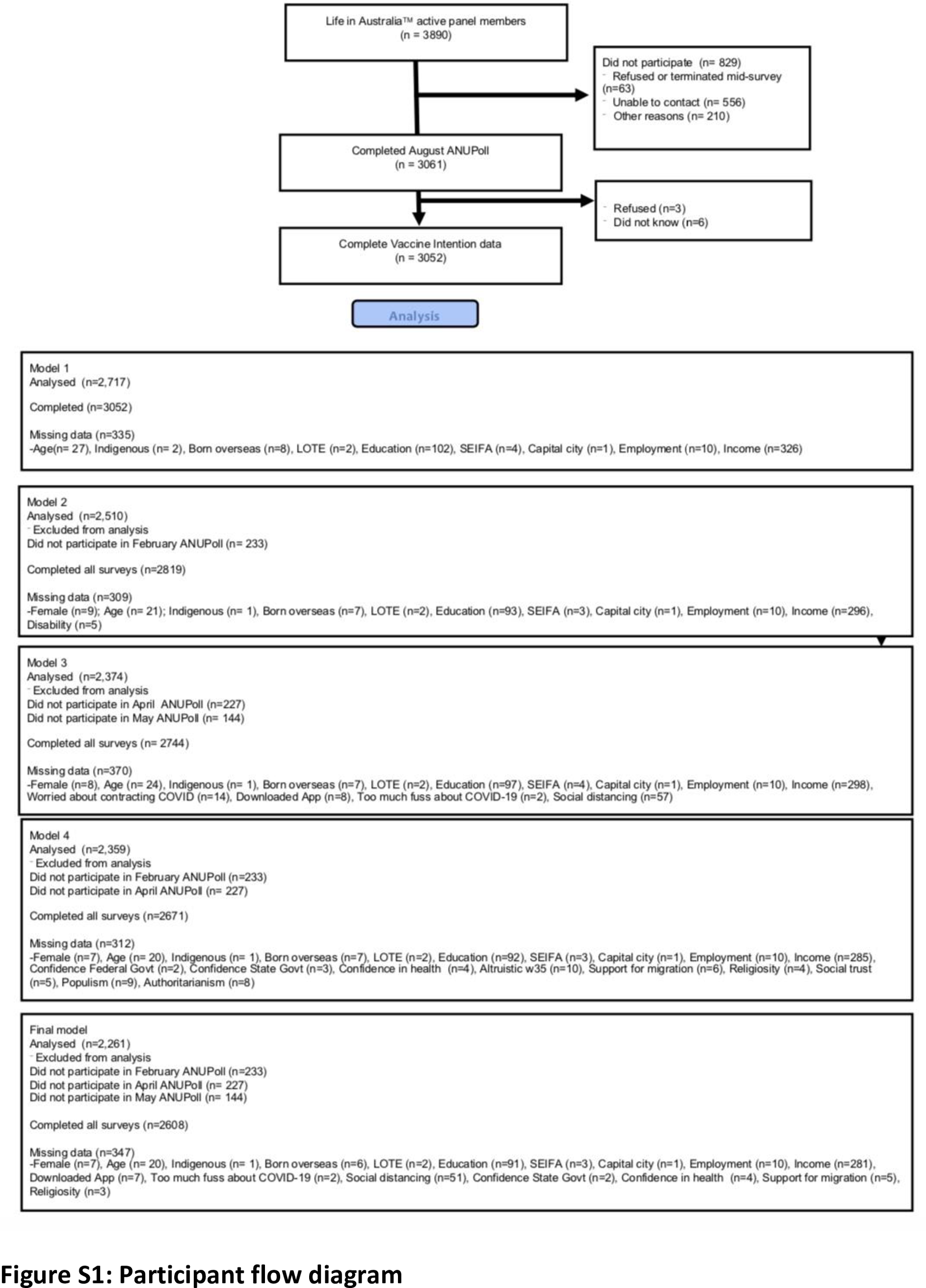

Supplement: S1 Fig — (TIF) [file pone.0248892.s001.tif]
