# Supplementary material for: COVID-19 vaccine hesitancy and resistance: Correlates in a nationally representative longitudinal survey of the Australian population
Source: PLoS One. 2021 Mar 24;16(3):e0248892. doi: 10.1371/journal.pone.0248892 (PMC7990228; doi:10.1371/journal.pone.0248892)
Supplement: Attachment — Submitted filename: Response to reviewers PONE-D-20-37174.docx [file pone.0248892.s003.docx]

**Manuscript title:** COVID-19 vaccine hesitancy and resistance: Correlates in a nationally representative longitudinal survey of the Australian population

**Manuscript ID:** PONE-D-20-37174

**Response to Editor and Reviewer comments**

Please find our specific responses to comments from the Editor and Reviewers. Thank you for your constructive comments and reviews, detailed responses are in the table below.

Row E1.5 refers to revising our statement on funding. Please include the following statement:

'The Australian Institute of Health and Welfare (AIHW) provided financial support for the collection of the August ANUpoll data presented in this paper.’

Regards

Ben

Australian National University

| **Ref. #** | **Comment** | **Response** | **Reference in revised manuscript** |
| --- | --- | --- | --- |
| **Editor comments** | | | |
| E1.1 | Please ensure that your manuscript meets PLOS ONE's style requirements, including those for file naming. The PLOS ONE style templates can be found at  https://journals.plos.org/plosone/s/file?id=wjVg/PLOSOne_formatting_sample_main_body.pdf and  https://journals.plos.org/plosone/s/file?id=ba62/PLOSOne_formatting_sample_title_authors_affiliations.pdf | We have reviewed PLOS ONE’s style requirements and edited the manuscripts appropriately. |  |
| E1.2 | In your Methods section, please provide additional information about the methodology used. Please clarify how participants were selected and recruited in the original survey; and whether you applied additional eligibility criteria for inclusion in this analysis (please provide a participant flowchart) .  Moreover, please ensure that all variables are defined in the Methods section, and that it is clear how these were coded for your analysis. | We have added additional information about how participants were selected and recruited in the original survey, and included a reference to more detailed recruitment and methodological information in the statistical analyses section:  Model 1 included demographic variables from survey respondents who completed the August 2020 wave of data collection and who had complete vaccination intention data and demographic characteristics (Fig S1). Model 2 included demographic and health variables with a measure of disability from the February 2020 ANU Poll. Model 3 included demographic and COVID-19 related variables from April and May 2020 ANUPoll. Model 4 included demographic and political and social attitudes from February and April 2020 ANUPoll. To understand the relative importance of the variables included in the models, Model 5 included demographic variables and statistically significant variables (p<0.05) from models 2-4 including variables from February, April and May ANUPoll. There was complete vaccine intention information for 3,052 participants. The number of participants in each of the models varied depending on the rate of survey completion in other ANUPolls and missing data for particular variables in the survey (Fig S1).  In terms of eligibility criterion for inclusion in the analysis, we have clarified this using a participant flowchart.  There are many variables included in our models, descriptions of all variables are in the Appendix that is referred to the Methods section.  We have also added more information on the coding of variables for our analysis. | Lines 216-228 page 8  See Fig S1  See Appendix pages 1 to 2  See Appendix pages 1 to 2 |
| E1.3 | Finally, please include additional information regarding the survey or questionnaire used in the study and ensure that you have provided sufficient details that others could replicate the analyses. For instance, if you developed a questionnaire as part of this study and it is not under a copyright more restrictive than CC-BY, please include a copy, in both the original language and English, as Supporting Information.  Moreover, please include more details on how the questionnaire was pre-tested, and whether it was validated. | We have provided a link to the questionnaire which can be downloaded, see  Data and copies of the questionnaire are available through the Australian Data Archive.^[1]^ | Line 174, page 7 |
| E1.4 | In the ethics statement in the Methods and online submission information, please ensure that you have specified (i) whether consent was informed and (ii) what type you obtained (for instance, written or verbal).  If your study included minors, state whether you obtained consent from parents or guardians.  If the need for consent was waived by the ethics committee, please include this information. | This has been updated: “Informed consent was provided online or verbally depending on the initial means of recruitment.” | Line 173-174, page 7 |
| E1.5 | Thank you for stating the following after the Conclusion Section of your manuscript:  'Funding The Australian Institute of Health and Welfare (AIHW) provided financial support for the analysis of the August ANUpoll data presented in this paper.'    We note that you have provided funding information that is not currently declared in your Funding Statement. However, funding information should not appear in the Acknowledgments section or other areas of your manuscript. We will only publish funding information present in the Funding Statement section of the online submission form.  a. Please remove any funding-related text from the manuscript and let us know how you would like to update your Funding Statement. Currently, your Funding Statement reads as follows:  'The survey data used in this paper were collected with support from the Australian Institute of Health and Welfare.  AIHW did not have any input to the manuscript.'  b. Please include your amended statements within your cover letter; we will change the online submission form on your behalf. | Thank you, we have provided an amended funding statement at the beginning of this cover letter | Cover letter |
| E1.6 | 5. Please include captions for your Supporting Information files at the end of your manuscript, and update any in-text citations to match accordingly. Please see our Supporting Information guidelines for more information: [http://journals.plos.org/plosone/s/supporting-information](about:blank) |  |  |
| **Reviewer comments** | | | |
| R1.1 | Introduction: delete line 55-68-84-102. Update data on COVID bruden worldwide at the day of resumbission | Deletions of lines 55-68-84-102 have been undertaken  Where appropriate we updated the data on COVID burden  We have also updated the references as some working papers have now been published in journals. |  |
| R1.2 | Discussion: add some future perspectives as take home message to improve and reduce the hesitancy and resistance and how is the role of doctors, journalist and politicians to improve it  The role of safety is also important (see and cite The efficacy and safety of influenza vaccination in older people: An umbrella review of evidence from meta-analyses of both observational and randomized controlled studies. Ageing Res Rev. 2020 Sep;62:101118. doi: 10.1016/j.arr.2020.101118. Epub 2020 Jun 18. PMID: 32565328.) | Thank you for your suggestion, we had included in our discussion the following but now highlight that doctors, journalists and politicians have a role (changes in bold) :  Pre-emptively using cognitive inoculation techniques and pre-bunking techniques [16] to actively work against the likely misinformation that will be generated in the development and preparation phase of a vaccine for COVID-19 will be important but probably requires more targeted and nuanced public health messages by trusted members of the community **including doctors, journalists and politicians**.  We agree that safety is important, thank you for your suggested reference. This has now been cited on page 13 | Line 349, page 13  Line 342, page 13 |
| R2.1 | The authors have conducted a survey to identify what the potential uptake will be for the COVID-19 vaccines and provided information on the demographics of the population who are most and least likely to be vaccinated. This is an important publication. I think that it might be useful to provide examples of how trust could be built for this vaccine/or has been built for other vaccines. Likewise despite the desperate situation that COVID-19 has put us in it might also be good to comment that it affords a lesson to develop new ways to reach difficult to vaccinate communities. | Thank you for your positive review, we appreciate your suggestions.  We have added the following to the discussion:  Other strategies to engender trust among the population could include enlisting community and cultural leaders to assist in the development and spread of information [19]. | Lines 349-351, page 13 |

1. https://dataverse.ada.edu.au/dataset.xhtml?persistentId=doi%3A10.26193%2FZFGFNE
